# Operations on the Insane

**Published:** 1897-10-02

**Authors:** 


### Operations on the Insane.

The attempt to influence the progress of insanity in women by operations on their generative organs involves so many and such serious questions, both in pathology and ethics, that we feel bound at once to draw attention to and to enter a protest against certain views which have recently been promulgated on the subject in America.

The matter is all the more serious from the fact that women are only too ready to accept the suggestion that their nervous derangements are due to some disorder of their pelvic organs.

They go by their own feelings, and since most women, by the time they are old enough to have to advise their daughters on such matters, have already discovered for themselves how largely their mental state varies with the crises through which they have to go, the ground is prepared for the dangerous doctrine that nervous derangements in women, even when culminating in the production of insanity, are to be dealt with by treatment directed to the generative organs.

This is a doctrine which has gone through many phases. In one form and another it has again and again cropped up, to be met sometimes with acceptance, sometimes with protest and objection; and, after many oscillations to one side and the other, opinion in England has at last fairly settled down to this position, that while it is justifiable to remove diseased organs healthy ones must not be interfered with, even on the chance of thereby relieving serious mental derangement.

On the face of it this would seem a reasonable conclusion. But behind it there is another side of the question which has been brought into disagreeable prominence by a discussion which took place on the subject at Montreal. What this is may be best indicated by asking the still further questions, What is a diseased organ, and what sort of disease will justify operative interference when the patient herself is mentally incompetent? It is here that the prevailing prejudice of women in favour of attributing mental disturbances to local disorders of the pelvic organs becomes of importance, because as a result of it a rash and enthusiastic operator can always obtain support and encouragement of a kind in proceedings which otherwise would meet only with reprobation. If women are ready, as they undoubtedly are, to submit their own persons to the knife for the relief of all sorts of imaginary ailments, how much more likely will they be to offer up their sisters on the chance of giving them back their reason? Even without this excuse we must all be aware how indifferent the friends of lunatics often are, and how ready they are to snatch at any suggestion which offers a hope of relief even though the risk may be great. A kill-or-cure operation is only too readily consented to by the friends of a patient who, as the saying goes, "is but a burden both to herself and others."

It is important then to inquire with what frequency an enthusiastic operator can find disease of the pelvic organs if he looks out for it, and how often he will operate if he has his own way. Certain papers read at Montreal give us the answers to these questions. One speaker, the superintendent of a large hospital for the insane, stated that in his own examinations "60 per cent. of the women had some abnormal condition of the pelvic organs, not always grave, often not calling for treatment; nevertheless, distinct pathological conditions easily recognised." At another place, however, "80 per cent. of the women admitted had local pelvic disease calling for treatment." Now we would be the last to say that because a woman was insane she should not have proper treatment for her local ailments. But to see what this line of investigation may lead men to we must turn to another paper read at the same meeting. We quote the exact words: "The gynæcological examination of 100 insane women pointed to the existence of pelvic disease in 93 patients; and in 89 of the 100 cases operative treatment was undoubtedly needed for the improvement of their physical condition." Then comes a list of the conditions found, and those for which operations were performed. Out of 80 cases reported, the following operations had been done: Divulsion and curettage, 14; repair or amputation of the diseased cervix in addition to curettage, 27; Alexander's operation for replacing the uterus, 8; ventri-suspension, 3; hysterectomy, 12 (!), 2 of whom died; removal of ovaries and tubes, 10, with 1 death; &c. We find it so difficult to believe that 12 cases requiring hysterectomy, and 10 requiring removal of their tubes and ovaries, could be found in any 100 women taken consecutively, that we can only hope that the reader of this paper has been misreported; but even taking the whole of the asylum from which this experience is gained—one having "a female population of nearly 600 patients"—and taking the whole two years and a half which have elapsed since "surgical gynæcology was introduced as a rational mode of treatment," we cannot but think that this is an appalling record.

Fortunately, some of the alienists who were present were able to throw a little cold water on the proposals of these enthusiastic gynæcologists. But the statements as to the beneficial effects of surgical interference were so positive, and the claim for the acceptance of this line of treatment were put forward in so formal and definite a manner, that we think they should be nailed down at once as bad coin, and as not admissible for a moment. Women in England, however, are not entirely unprotected; and Dr. Urquhart, in speaking against these wild proposals, very properly pointed out that, on this side of the water, it was one of the risks of the profession that one might have to justify an operation before judge and jury.

